# Supplementary figures and images for: An in vivo drug repurposing screen and transcriptional analyses reveals the serotonin pathway and GSK3 as major therapeutic targets for NGLY1 deficiency
Source: PLoS Genet. 2022 Jun 2;18(6):e1010228. doi: 10.1371/journal.pgen.1010228 (PMC9162339; doi:10.1371/journal.pgen.1010228)

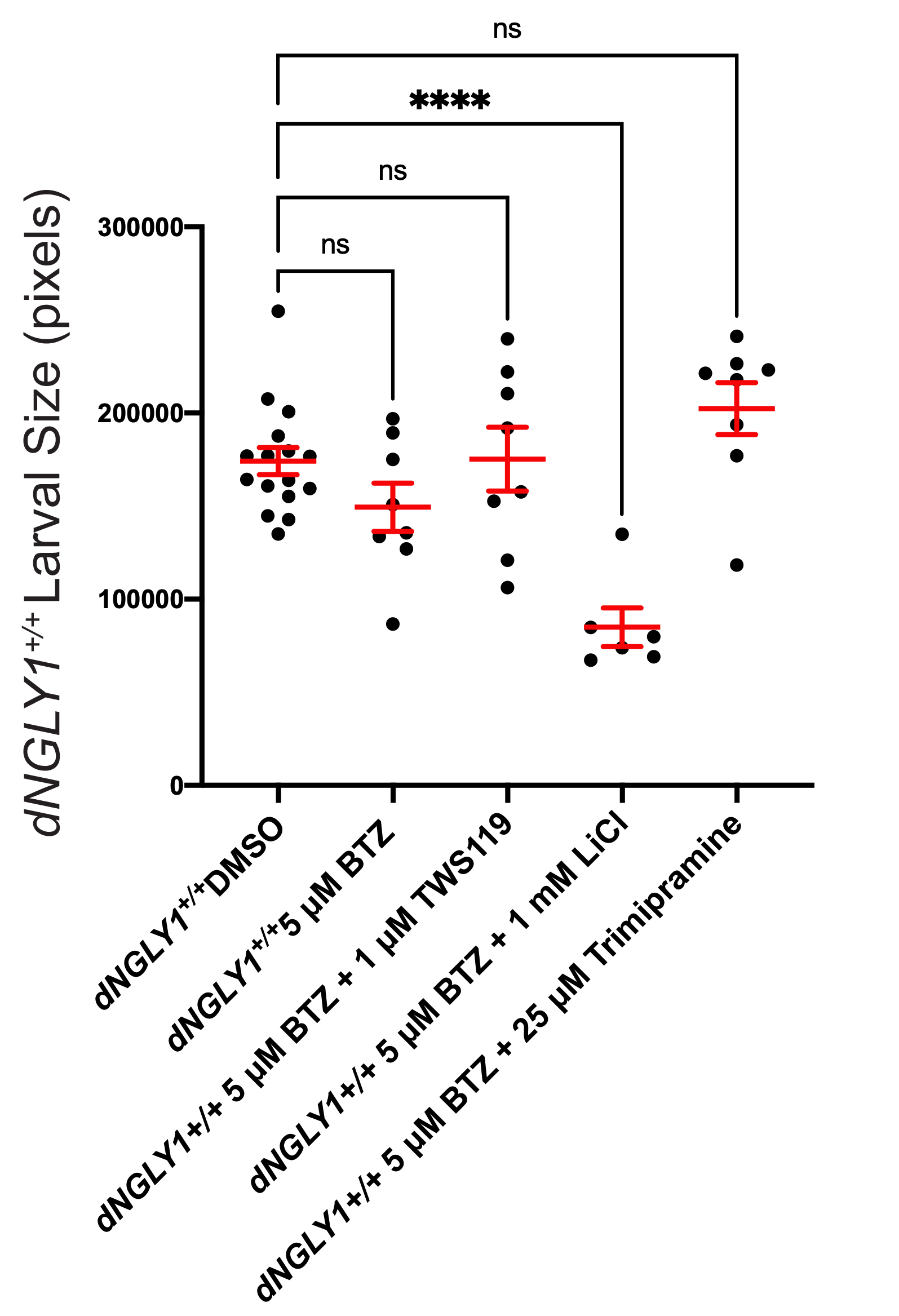

Supplement: S1 Fig — There was no effect of treatment on larval size, except for BTZ + LiCl, where larvae were significantly smaller than DMSO treated controls. ****p < 0.0001. (PNG) [file pgen.1010228.s001.png]
